# *Notes from the Field*: Multistate Outbreak of *Escherichia coli* O157:H7 Infections Linked to Dough Mix — United States, 2016

**DOI:** 10.15585/mmwr.mm6603a6

**Published:** 2017-01-27

**Authors:** Laura Gieraltowski, Colin Schwensohn, Stephanie Meyer, Dana Eikmeier, Carlota Medus, Alida Sorenson, Matthew Forstner, Asma Madad, Joseph Blankenship, Peter Feng, Ian Williams

**Affiliations:** ^1^National Center for Emerging and Zoonotic Infectious Diseases, CDC; ^2^Minnesota Department of Health; ^3^Minnesota Department of Agriculture; ^4^Food and Drug Administration.

On January 4, 2016, CDC PulseNet, the molecular subtyping network for foodborne disease surveillance, identified a cluster of 10 Shiga toxin-producing *Escherichia coli* (STEC) O157:H7 infections with indistinguishable pulsed-field gel electrophoresis (PFGE) pattern combinations. STEC infections with the identified outbreak PFGE pattern are commonly reported to PulseNet, with an average of 40–50 illnesses reported annually. Because this was a relatively common strain of STEC, multiple locus variable-number tandem repeat analysis (MLVA), another subtyping technique used to characterize the genetic relatedness of bacteria, was used to help define cases in the cluster. CDC collaborated with state and local health and agricultural agencies and the Food and Drug Administration (FDA) to investigate the outbreak. A case was defined as STEC O157:H7 infection with an isolate having PFGE and MLVA patterns indistinguishable from the outbreak strain in a person with diarrheal illness onset during December 6, 2015–February 9, 2016.

Thirteen STEC O157:H7 outbreak-associated cases were identified in nine states: Iowa (one case), Illinois (one), Kansas (one), Minnesota (five), North Carolina (one), Nebraska (one), New Jersey (one), South Dakota (one), and Wisconsin (one). The median age of patients was 17 years (range = 7–71 years); 53% were female. Among 12 patients with available information, eight were hospitalized, including two who developed hemolytic uremic syndrome; no deaths were reported.

Among the 12 interviewed patients, nine reported eating at one of nine locations of restaurant A, a national restaurant chain, during the week preceding illness onset, including eight who ate a specific dessert pizza made with a proprietary dough mix provided by manufacturer A. The ninth patient consumed bread sticks made from the same dough mix. At one Minnesota location, six of 21 (28%) non-ill patrons reported eating the implicated dessert pizza. Assuming this was representative of patrons of restaurant A, the proportion of cases who consumed dessert pizza was significantly higher than what would be expected by chance using the binomial distribution model (p<0.001). As an intervention in this outbreak, restaurant A locations stopped using dough mix from manufacturer A on February 4, 2016.

Eighty-eight samples of dry dough mix from five restaurant A locations where patients reported eating were collected by public health officials in five states (Illinois, Iowa, Minnesota, Nebraska, and Wisconsin). The Minnesota Department of Agriculture identified non-O157 STEC in seven of 17 collected samples, including one Shiga toxin-1–producing non-O157 STEC isolate and six Shiga toxin-2 (stx2)–producing non-O157 STEC isolates. FDA collected six samples of dry dough mix from manufacturer A. All six samples tested negative for STEC O157:H7, but one yielded an stx2-producing STEC O8:H28. All identified strains lacked known adherence factors and were therefore considered to present a low health risk.

Flour is a raw agricultural product and does not undergo processing to kill bacteria and other pathogens, so it is not sterile. Generic *E. coli* and coliforms have been found previously in flour (*1*,*2*). Flour and flour-based mixes have been suspected or implicated as the source of other foodborne *Salmonella* and STEC O157 outbreaks (*1*,*3*–*6*). Of note, this PFGE pattern was previously isolated from a sample of bulk flour collected during a 2009 outbreak investigation (*5*). Although no laboratory evidence identified contaminated flour as the ultimate source of this STEC O157:H7 outbreak, the identification of other enteric pathogens in multiple samples of dry dough mix consumed by patients associated with the outbreak implicates contaminated flour as the possible source of pathogen introduction for this outbreak. The small number of cases and the lack of additional restaurant clusters suggest that this was a low level contamination event or that contamination only affected a limited amount of product. Evidence obtained at one restaurant A location showed that dessert pizzas were made with the same dough mix used in traditional pizzas, but used thicker dough and might have been undercooked at some locations. Flour is usually not thought to be a food safety risk, but flour-based mixes are ubiquitous in restaurants and are often used for dusting of surfaces for transfer of pizzas. This outbreak serves as a reminder that consumers, industry, and government should consider that flour, a raw agricultural product, might be contaminated with pathogens and, when consumed raw or undercooked, might pose a risk to human health.
